# Combined association between dietary inflammatory index-related dietary patterns and physical activity with frailty in Chinese older adults: findings from a cross-sectional

**DOI:** 10.3389/fpubh.2025.1739530

**Published:** 2026-01-12

**Authors:** Huatao Zheng, Dan Li, Rentao Ma, Zihao Huang, Qingyun Ding, Shiqiang Wang

**Affiliations:** 1 Hunan University of Technology, Zhuzhou, China; 2 Hunan Provincial Key Laboratory of Physical Health and Fitness, Zhuzhou, China

**Keywords:** combined effects, dietary patterns, older adults, frailty, physical activity

## Abstract

**Background:**

With the aging population in China, research on preventing frailty is crucial. This study aims to investigate the independent and combined associations of the Dietary inflammatory index (DII) and physical activity (PA) with frailty among Chinese older adults.

**Methods:**

A total of 285 participants aged ≥60 years with 87 males and 186 females were recruited from Hunan Province. Daily moderate physical activity (MPA), vigorous physical activity (VPA) and light physical activity (LPA) were objectively measured using a triaxial accelerometer. A Food Frequency Questionnaire 25 (FFQ25) was used to assess the participants’ dietary patterns, and DII was calculated. Six combined exposure groups were formed based on PA and DII: pro-inflammatory diet and insufficient PA group, neutral diet and insufficient PA group, anti-inflammatory diet and insufficient PA group, pro-inflammatory diet and sufficient PA group, neutral diet and sufficient PA group, and anti-inflammatory diet and sufficient PA group. Frailty was assessed using the Frailty Phenotype (FP), logistic regression analyzed the associations between dietary patterns, PA, and frailty.

**Results:**

A total of 285 older adults participants were initially recruited, but 12 were excluded due to missing data. Consequently, 273 participants were included in the final analysis. Compared to individuals with insufficient PA, those with sufficient PA were associated with significantly lower odds of frailty (OR = 0.468, 95%CI = 0.242–0.907). Participants following an anti-inflammatory diet had significantly lower odds of frailty compared with those following a pro-inflammatory diet (OR = 0.467, 95%CI = 0.221–0.988). In the combined groups, frailty prevalence was significantly lower the group with anti-inflammatory diet and sufficient PA group (OR = 0.204, 95%CI = 0.072–0.583), compared with pro-inflammatory diet and insufficient PA group. The sensitivity analysis showed that the associations between anti-inflammatory diet and sufficient PA with frailty remained statistically significant, with the direction of the associations unchanged. These findings suggest that the results are robust.

**Conclusion:**

Our study indicates that adhering to an anti-inflammatory diet and maintaining sufficient PA may be associated with a lower likelihood of frailty. Achieving an adequate amount of PA and following a healthy dietary pattern may serve as potential preventive measures against frailty.

## 1 Introduction

With the increasing aging population, older adults health issues are drawing widespread global attention. Among these, frailty, as a comprehensive indicator reflecting the overall health status of older adults, directly affects their daily living functions and independence. Frailty manifests as a decline in the physiological reserves of multiple systems and a reduced tolerance to stress, which can lead to various complications such as cognitive decline, cardiovascular diseases, and chronic illnesses. This increases the risk of falls, disability, hospitalization, and even death in older adults (1). A report covering 62 countries worldwide indicates that the frailty prevalence in community-dwelling older adults has reached 12% or even higher (2). Data show that the frailty rate among community-dwelling older adults in China ranges from 5.9 to 17.4% (3). Furthermore, as age increases and unhealthy lifestyles persist, the incidence of frailty may rise further (4). If frailty is not addressed early, it can severely impact the quality of life in older age, reduce healthy life expectancy, and potentially lead to a series of social issues, such as the excessive consumption of healthcare resources and an increased burden on families and society. Therefore, early scientific intervention in frailty is of paramount importance.

Previous studies have shown (5) that interventions such as nutritional support and physical activity (PA) can significantly reduce the risk of frailty and contribute to its positive progression. Achieving the PA levels recommended by the World Health Organization (WHO) may help prevent and intervene in frailty through mechanisms such as enhancing physical function, improving metabolic health, and delaying the onset of chronic diseases (6). Several studies have explored the relationship between PA and frailty. For instance, Ma et al. used data from the 4th wave of CHARLS and found that high levels of PA significantly reduced frailty risk across different age groups (7). The frailty incidence rate in those with high activity levels was approximately 0.95 times that of those with low activity levels. Fung et al., in a study of 1992 older adult women in the United States, found that moderate-to-high PA was associated with a lower risk of frailty (8). On the other hand, nutritional interventions, particularly healthy dietary patterns, have also been shown to reduce frailty incidence (9, 10). Previous studies have demonstrated that individuals following a pro-inflammatory diet may be at a higher risk of frailty or pre-frailty. In contrast, an anti-inflammatory diet, rich in polyphenols and dietary fiber, directly suppresses inflammation markers, reduces pro-inflammatory factors, and thus mitigates frailty (11). A study conducted in China revealed that the frailty incidence among individuals following a pro-inflammatory diet was 1.82 times higher than those on an anti-inflammatory diet (12). In a study by Kim et al., based on data from 321 older adults in South Korea, a significant positive correlation was found between the dietary inflammation index and frailty scores, with individuals following a pro-inflammatory diet performing worse on frailty tests (13).

Although existing evidence has established the independent protective roles of PA and healthy dietary patterns in preventing frailty, research on their combined effect among Chinese older adults remains limited. PA and an anti-inflammatory diet may concurrently target the core process of “inflammaging.” By mitigating the detrimental effects of chronic inflammation and oxidative stress on multisystem physiological function, these two behaviors may produce cumulative or synergistic effects at molecular and systemic levels, thereby more effectively preserving physiological reserves and preventing the onset of frailty. However, most previous studies have focused on these factors in isolation, with little attention given to their combined association. Therefore, systematically evaluating the independent and combined associations of PA and dietary patterns with frailty, using empirical data from a Chinese older adult population, is crucial. Such an investigation will not only help elucidate the complex mechanisms through which behavioral factors influence healthy aging but also provide a key scientific foundation for developing integrated and effective strategies to prevent frailty.

## 2 Materials and methods

### 2.1 Participants

Convenience sampling was used to recruit participants from several communities in the Xianyueshan Street area of Liling City, Hunan Province, including the Nanmen, Shuyuan, and Bishan communities. The recruitment period for this study began on June 5, 2025, and ended on July 15, 2025. The inclusion criteria for participants were as follows: (1) aged 60 years or older; (2) no significant physical disabilities, with the ability to perform normal PA; (3) willingness to sign written informed consent. A total of 285 older adult individuals aged 60 and above were recruited. Trained investigators conducted surveys with the participants, including collecting basic demographic information, frailty assessment, dietary pattern questionnaires, and assisting participants in wearing accelerometers to measure PA. 12 participants were excluded from the final analysis: 5 for insufficient accelerometer wear days or duration, 2 for invalid FFQ25 questionnaires, and 5 for missing frailty assessment data. The sample size for this study was determined *a priori* using the widely adopted rule of thumb for prediction models, which requires a minimum of 10 events per variable (EPV) (14, 15). With six independent variables and an anticipated frailty prevalence of 25%, this study required a minimum of 60 events. The sample size estimation was based on an anticipated frailty prevalence of 25%. This value is higher than the rates generally reported in community-based studies, reflecting a conservative estimation strategy adopted to account for the higher average age of the older adult population included in this study and to ensure sufficient statistical power. This corresponded to a minimum total sample size of 240 participants. To account for potential participant attrition or missing data, an inflation of 10% was applied, setting the initial recruitment target for this study to 267 participants. Ultimately, 273 participants were enrolled, exceeding our target and ensuring that the final model was developed with sufficient statistical power. All participants provided written informed consent. Approval for the studies involving human participants was obtained from the Research Ethics Management Panel of the Scientific Research Integrity and Ethics Committee, Hunan University of Technology (No. SK2025-004).

### 2.2 Frailty

Frailty was assessed using the Fried frailty phenotype (FP) (16). The specific criteria for assessment were as follows: (1) Unintentional weight loss: Weight loss of ≥5% or ≥4.5 kg in the past year; (2) Low grip strength: Grip strength of the dominant hand was measured using a hand-held dynamometer. For males, grip strength was classified based on body mass index (BMI): BMI ≤ 24, grip strength ≤ 29 kg; 24 < BMI ≤ 28, grip strength ≤ 30 kg; BMI > 28, grip strength ≤ 32 kg. For females, grip strength was classified based on BMI: BMI ≤ 23, grip strength ≤ 17 kg; 23 < BMI ≤ 26, grip strength ≤ 17.3 kg; 26 < BMI ≤ 29, grip strength ≤ 18 kg; BMI > 29, grip strength ≤ 21 kg. Individuals meeting these criteria were classified as having low grip strength, while those who did not were classified as having normal grip strength; (3) Self-reported fatigue: The CES-D scale items, “In the past week, I felt that doing anything required a lot of effort” and “I could not get going,” were used. If any of the responses were “occasionally (3–4 days)” or “most of the time (5–7 days),” the individual was classified as fatigued; (4) Slow walking speed: Walking speed was measured over a 4.57-meter distance. For males, walking time was classified by height: ≤173 cm, time ≥7 s; >173 cm, time ≥6 s. For females, walking time was classified by height: ≤159 cm, time ≥7 s; >159 cm, time ≥6 s. Individuals meeting these criteria were classified as having low walking speed, while those who did not were classified as having normal walking speed; (5) Low PA: The International Physical Activity Questionnaire Short Form (IPAQ-SF) was used to assess PA levels. Individuals with a total weekly PA level of less than 600 metabolic equivalent minutes (MET-min/wk) were classified as having low PA. Participants who met three or more of these criteria were classified as “frail,” those meeting one or two criteria as “pre-frail,” and those meeting none as “non-frail.”For the purposes of this study, participants categorized as “no frailty” (including those in the pre-frailty category) were grouped together and compared to those classified as “frail.”

### 2.3 Physical activity

PA was measured using a triaxial accelerometer (Actigraph wGT3X-BT, Pensacola, FL, USA). Participants were instructed to wear the accelerometer continuously for 7 days, except when sleeping, bathing, or swimming. During this period, they were asked to maintain their usual daily activities (17). The 7-day wear period included both weekdays and weekends. Research staff conducted regular follow-ups via phone to remind participants to wear the accelerometer as instructed, ensuring the completeness and validity of the data. The raw accelerometer data had a sampling frequency of 30 samples per second (30 Hz) and were imported into Actilife software version 6.13.4 for processing, with data converted into 60-s intervals. Valid data were defined as wearing the accelerometer for at least 10 h per day, with at least 4 valid days of wear. There were no restrictions regarding whether the valid days included weekdays or weekends. Non-wear time was defined as at least 90 min of consecutive zero counts, allowing a maximum of 2 min with counts less than 100 counts per minute. LPA and Moderate-to-Vigorous PA MVPA were categorized based on Freedson’s cutoff points, where LPA was defined as 100–1951 counts per minute, and MVPA was defined as ≥1952 counts per minute (18). For older adult people who have been wearing accelerometers for 4 or more effective days, calculate the daily MVPA activity time based on the average MVPA duration over the 4 days. According to the WHO’s definition (19), PA was classified as “sufficient PA” if: (1) for adults aged 18–64, they engaged in at least 150–300 min of moderate-intensity aerobic activity, or at least 75–150 min of vigorous-intensity aerobic activity, or an equivalent combination of both; (2) for individuals aged 65 and above, they engaged in at least 150–300 min of moderate-intensity aerobic activity, or at least 75–150 min of vigorous-intensity aerobic activity, or an equivalent combination of both. PA that did not meet these conditions was classified as “insufficient PA.”

### 2.4 Dietary pattern

Dietary intake over the past year was collected using the FFQ25 questionnaire, which assesses the frequency of consumption of 25 food items. The frequency categories ranged from “never” to “three or more times per day,” with corresponding weights of 0.00, 0.03, 0.07, 0.22, 0.50, 0.79, 1.00, 2.00, and 3.00. The portion sizes were categorized from “less than 50 g” to “greater than 250 g,” with weights of 0.50, 0.75, 1.00, 1.50, and 2.00, respectively. Special food items such as eggs, beverages, and beer were measured in specific units, such as individual pieces or bottles. The FFQ25 has been tested and shown to have high reliability and good validity (20). DII was developed by researchers at the University of South Carolina as part of the Cancer Prevention and Control Program to assess the overall inflammatory potential of an individual’s diet, the DII has been tested and shown to have high reliability and good validity (21). It is calculated by comparing the individual’s dietary intake data with the global average intake of each food component. For this study, the DII was based on the 25 food items in the FFQ25 and calculated using 14 dietary components, including energy, protein, fat, carbohydrates, dietary fiber, cholesterol, vitamins A, B1, B2, C, E, calcium, iron, and zinc. First, calculate the individual’s total daily energy and nutrient intake. Then, compare it with the global average intake and standard deviation to calculate the individual’s Z-score: Z = (individual’s average daily intake of dietary component - global average intake) / global average intake standard deviation. Next, convert the Z-score into a percentile value, double the percentile value, and then subtract 1 for centering. Finally, multiply the value obtained in step 3 by the corresponding inflammatory response score for the dietary component to obtain the individual’s inflammatory effect score for each dietary component. The Dietary Inflammatory Index (DII) is obtained by summing the inflammatory scores for all components. The DII was then categorized into three groups based on tertiles: anti-inflammatory diet group, neutral diet group, and pro-inflammatory diet group (22).

### 2.5 Covariates

Demographic and lifestyle information, including age, gender, residential area, educational level, marital status, smoking status, and alcohol drinking, was collected using a standardized questionnaire. Height and weight were objectively measured by trained staff using calibrated instruments, and BMI was calculated based on these measurements.

### 2.6 Statistical analyses

Data were organized using Stata 17.0 statistical software, and to examine the individual and combined associations of PA and dietary patterns with frailty, we performed separate and combined binary logistic regression analyses, along with descriptive and sensitivity analyses, using SPSS version 27.0. Categorical variables were expressed as proportions and rates, and between-group comparisons were performed using the Chi-square (χ^2^) test. A multivariable unconditional logistic regression model was used to analyze the independent effects of PA and dietary patterns on frailty in the older adults. Six combined groups were formed based on PA and dietary patterns: pro-inflammatory diet and insufficient PA group; neutral diet and insufficient PA group; anti-inflammatory diet and insufficient PA group; pro-inflammatory diet and sufficient PA group; neutral diet and sufficient PA group; and anti-inflammatory diet and sufficient PA group. A multivariable unconditional logistic regression model was used to assess the combined effects of PA and dietary patterns on frailty. A sensitivity analysis was performed after excluding older adult individuals aged 80 years and older. Multiplicative and additive interaction models assessed the interaction effects between dietary patterns and PA. Additive and multiplicative interaction analyses were performed using R software. Multiplicative interaction was evaluated by constructing multivariable logistic regression models that included an interaction term between dietary patterns and PA, with adjustment for relevant confounding factors. Additive interaction was assessed by calculating the relative excess risk due to interaction (RERI), attributable proportion (AP), and synergy index (S). Statistical significance for multiplicative interaction was considered when the *p*-value for the interaction term was <0.05. Additive interaction was established if the 95% confidence intervals for both RERI and AP excluded zero, and simultaneously, the 95% CI for S excluded one. The results are presented as odds ratios (OR) with 95% confidence intervals (95% CI). A two-tailed significance level of *α* = 0.05 was used for all statistical tests.

## 3 Results

### 3.1 Sample characteristics

The final analysis included 273 older adult individuals with an average age of 71.6 ± 7.2 years, including 87 males (31.9%) and 186 females (68.1%). Among them, 33 participants (12.1%) lived in rural areas. Regarding education level, 118 participants (43.2%) had completed secondary school or lower. Regarding body mass index (BMI), 171 individuals (62.6%) had a normal BMI. For PA, 65 participants (23.8%) were classified as insufficiently active, while 208 participants (76.2%) met the recommended PA levels. Regarding dietary patterns, 91 participants (33.3%) followed a pro-inflammatory diet, 91 (33.3%) followed a neutral diet, and 91 (33.3%) adhered to an anti-inflammatory diet. Frailty status was as follows: 63 participants (23.1%) were frail, and 210 participants (76.9%) were no frailty. Statistically significant differences were observed between groups based on age, living area, PA status, and dietary patterns (*p* < 0.05) (Table 1).

**Table 1:** Baseline characteristics of the study population.

Variables	Dietary pattern and PA group, *n* (%)							*X^2^*	*p*
	Total	Pro-inflammatory diet and insufficient PA	Neutral diet and insufficient PA	Anti-inflammatory diet and insufficient PA	Pro-inflammatory diet and sufficient PA	Neutral diet and sufficient PA	Anti-inflammatory diet and sufficient PA		
Age group								18.39	0.002
60-74 years	181 (66.3)	11 (42.3)	16 (59.3)	5 (41.7)	43 (66.2)	53 (82.8)	53 (67.1)		
≥75 years	92 (33.7)	15 (57.7)	11 (40.7)	7 (58.3)	22 (33.8)	11 (17.2)	26 (32.9)		
Gender								2.76	0.736
Male	87 (31.9)	9 (34.6)	9 (33.3)	4 (33.3)	18 (27.7)	25 (39.1)	22 (27.8)		
Female	186 (68.1)	17 (65.4)	18 (66.7)	8 (66.7)	47 (72.3)	39 (60.9)	57 (72.2)		
Residence area								18.34	0.003
Rural	33 (12.1)	0 (0.0)	1 (3.7)	0 (0.0)	4 (6.2)	15 (23.4)	13 (16.5)		
Urban	240 (87.9)	26 (100.0)	26 (96.3)	12 (100.0)	61 (93.8)	49 (76.6)	66 (83.5)		
Education level								7.39	0.688
Below middle school	118 (43.2)	11 (42.3)	8 (29.6)	5 (41.7)	26 (40.0)	30 (46.9)	38 (48.1)		
Middle school	142 (52.0)	14 (53.8)	19 (70.4)	6 (50.0)	34 (52.3)	32 (50.0)	37 (46.8)		
Above middle school	13 (4.8)	1 (3.8)	0 (0.0)	1 (8.3)	5 (7.7)	2 (3.1)	4 (5.1)		
Marital status								4.11	0.533
Without partner	81 (29.7)	8 (30.8)	7 (25.9)	6 (50.0)	20 (30.8)	21 (32.8)	19 (24.1)		
With partner	192 (70.3)	18 (69.2)	20 (74.1)	6 (50.0)	45 (69.2)	43 (67.2)	60 (75.9)		
Smoking status								7.95	0.159
No smoker	236 (86.4)	21 (80.8)	21 (77.8)	12 (100.0)	61 (93.8)	55 (85.9)	66 (83.5)		
Smoker	37 (13.6)	5 (19.2)	6 (22.2)	0 (0.0)	4 (6.2)	9 (14.1)	13 (16.5)		
Alcohol drinking								5.39	0.369
Non drinker	260 (95.2)	26 (100.0)	26 (96.3)	12 (100.0)	63 (96.9)	58 (90.6)	75 (94.9)		
Drinker	13 (4.8)	0 (0.0)	1 (3.7)	0 (0.0)	2 (3.1)	6 (9.4)	4 (5.1)		
BMI category								5.19	0.878
Healthy weight	171 (62.6)	18 (69.2)	18 (66.7)	7 (58.3)	37 (56.9)	36 (56.3)	55 (69.6)		
Overweight	60 (22.0)	5 (19.2)	6 (22.2)	3 (25.0)	15 (23.1)	16 (25.0)	15 (19.0)		
Obesity	42 (15.4)	3 (11.5)	3 (11.1)	2 (16.7)	13 (20.0)	12 (18.8)	9 (11.4)		
PA								273	<0.001
Insufficient	65 (23.8)	26 (100.0)	27 (100.0)	12 (100.0)	0 (0.0)	0 (0.0)	0 (0.0)		
Sufficient	208 (76.2)	0 (0.0)	0 (0.0)	0 (0.0)	65 (100.0)	64 (100.0)	79 (100.0)		
Dietary pattern								546	<0.001
Pro-inflammatory diet	91 (33.3)	26 (100.0)	0 (0.0)	0 (0.0)	65 (100.0)	0 (0.0)	0 (0.0)		
Neutral diet	91 (33.3)	0 (0.0)	27 (100.0)	0 (0.0)	0 (0.0)	64 (100.0)	0 (0.0)		
Anti-inflammatory diet	91 (33.3)	0 (0.0)	0 (0.0)	12 (100.0)	0 (0.0)	0 (0.0)	79 (100.0)		
Frailty status								14.74	0.011
No frailty	210 (76.9)	13 (50.0)	19 (70.4)	9 (75.0)	50 (76.9)	52 (81.2)	67 (84.8)		
Frailty	63 (23.1)	13 (50.0)	8 (29.6)	3 (25.0)	15 (23.1)	12 (18.8)	12 (15.2)		

### 3.2 Association of dietary patterns and PA with frailty

We first examined the associations of dietary patterns and PA with frailty status. In the unadjusted analysis (Model 1), the prevalence of frailty was significantly lower among participants with sufficient PA compared to those with insufficient PA (OR = 0.394, 95% CI = 0.217–0.727). Similarly, adherence to an anti-inflammatory diet was associated with a lower likelihood of frailty compared to adherence to a pro-inflammatory diet (OR = 0.444, 95% CI = 0.218–0.904). After adjusting for age, gender, residence area, education level, and marital status (Model 2), the significant inverse association persisted, with participants having sufficient PA maintaining significantly lower odds of frailty (OR = 0.484, 95% CI = 0.255–0.920). After further adjustments for smoking, alcohol drinking, and BMI (Model 3), sufficient PA remained associated with significantly lower odds of frailty (OR = 0.468, 95% CI = 0.242–0.907), and the anti-inflammatory diet was associated with a lower likelihood of frailty (OR = 0.467, 95% CI = 0.221–0.988). However, no protective effect was observed for the neutral diet (Table 2).

**Table 2:** Logistic regression analysis of the association of dietary patterns and PA with frailty.

Group	Model 1		Model 2		Model 3	
	OR (95% CI)	*p*	OR (95% CI)	*p*	OR (95% CI)	*p*
Dietary pattern						
Pro-inflammatory diet	1 (ref)		1 (ref)		1 (ref)	
Neutral diet	0.634 (0.325–1.234)	0.18	0.707 (0.352–1.419)	0.329	0.624 (0.303–1.287)	0.202
Anti-inflammatory diet	0.444 (0.218–0.904)	0.025	0.493 (0.238–1.020)	0.057	0.467 (0.221–0.988)	0.046
PA						
Insufficient	1 (ref)		1 (ref)		1 (ref)	
Sufficient	0.394 (0.217–0.727)	0.003	0.484 (0.255–0.920)	0.027	0.468 (0.242–0.907)	0.024

^a^Model 1 was unadjusted. Model 2 adjusted for age, gender, residence area, education level, and marital status. Model 3 adjusted for age, gender, residence area, education level, marital status, smoking, alcohol drinking, and BMI.

### 3.3 Analysis of the interaction between dietary patterns and PA on frailty

This study evaluated the combined effects of dietary patterns and PA on frailty using multiplicative and additive interaction models. The results indicated that no significant multiplicative interaction between overall dietary patterns and physical activity was observed. But on the multiplicative scale, a significant interaction was observed between an anti-inflammatory diet and sufficient PA (*p* < 0.05), whereas no significant interaction was found between a neutral diet and sufficient PA. On the additive scale, the relative excess risk due to interaction (RERI) values for the combination of a neutral diet and sufficient PA and an anti-inflammatory diet and sufficient PA were 0.510 (95% CI: −0.586–0.902) and 0.546 (95% CI: −0.850–0.943), respectively, with both 95% confidence intervals including 0. In addition, the 95% confidence intervals for the synergy index (S) for both groups included 1. Based on the criterion that a significant additive interaction exists if the 95% CIs for RERI and the attributable proportion due to interaction (AP) do not include 0, or if the 95% CI for S does not include 1, it was concluded that no significant additive interaction was observed between any dietary pattern and PA (Table 3).

**Table 3:** Interaction between dietary patterns and PA on frailty.

Dietary pattern	PA	Multiplicative interaction		Additive interaction		
		OR (95%CI)	*p*	RERI (95% CI)	AP (95% CI)	S (95% CI)
Dietary pattern	PA	–	0.116			
Neutral diet	Sufficient	0.538 (0.259–1.119)	0.097	0.510(−0.586–0.902)	2.209(−1.710–8.416)	0.601 (0.144–1.937)
Anti-inflammatory diet	Sufficient	0.418 (0.203–0.858)	0.018	0.546 (−0.850–0.943)	3.047 (−3.500–11.287)	0.601 (−0.411–2.094)

### 3.4 Combined associations of PA and dietary patterns with frailty

We examined the combined effect of dietary patterns and PA by categorizing participants into six groups: (1) pro-inflammatory diet and insufficient PA, (2) neutral diet and insufficient PA, (3) anti-inflammatory diet and insufficient PA, (4) pro-inflammatory diet and sufficient PA, (5) neutral diet and sufficient PA, and (6) anti-inflammatory diet and sufficient PA. The results showed a significant interaction between dietary patterns and PA in relation to frailty. After adjusting for age, gender, residence area, education level, marital status smoking, alcohol drinking, and BMI (Model 3), the pro-inflammatory diet and sufficient PA group (OR = 0.321, 95% CI = 0.115–0.899), the neutral diet and sufficient PA group (OR = 0.241, 95% CI = 0.078–0.737), and the anti-inflammatory diet and sufficient PA group (OR = 0.204, 95% CI = 0.072–0.583) all showed significantly lower odds of frailty compared to the pro-inflammatory diet and insufficient PA group. In contrast, the neutral diet and insufficient PA group (OR = 0.375, 95% CI = 0.115–1.223) and pro-inflammatory diet and insufficient PA group (OR = 0.303, 95% CI = 0.061–1.496) showed no significant association with frailty (Table 4).

**Table 4:** Logistic regression analysis of the combined association of dietary patterns and PA with frailty.

Group	Model 1		Model 2		Model 3	
	OR (95% CI)	*p*	OR (95% CI)	*p*	OR (95% CI)	*p*
Dietary pattern and PA group						
Pro-inflammatory diet and insufficient PA	1 (ref)		1 (ref)		1 (ref)	
Neutral diet and insufficient PA	0.421 (0.136–1.301)	0.133	0.400 (0.126–1.266)	0.119	0.375 (0.115–1.223)	0.104
Anti-inflammatory diet and insufficient PA	0.333 (0.073–1.518)	0.156	0.320 (0.068–1.505)	0.149	0.303 (0.061–1.496)	0.143
Pro-inflammatory diet and sufficient PA	0.300 (0.115–0.784)	0.014	0.326 (0.121–0.882)	0.027	0.321 (0.115–0.899)	0.031
Neutral diet and sufficient PA	0.231 (0.086–0.623)	0.004	0.290 (0.100–0.842)	0.023	0.241 (0.078–0.737)	0.013
Anti-inflammatory diet and sufficient PA	0.179 (0.067–0.479)	<0.001	0.218 (0.079–0.603)	0.003	0.204 (0.072–0.583)	0.003

^a^Model 1 was unadjusted. Model 2 adjusted for age, gender, residence area, education level, and marital status. Model 3 adjusted for age, gender, residence area, education level, marital status, smoking, alcohol drinking, and BMI.

### 3.5 Subgroup analyses

**Figure 1:**
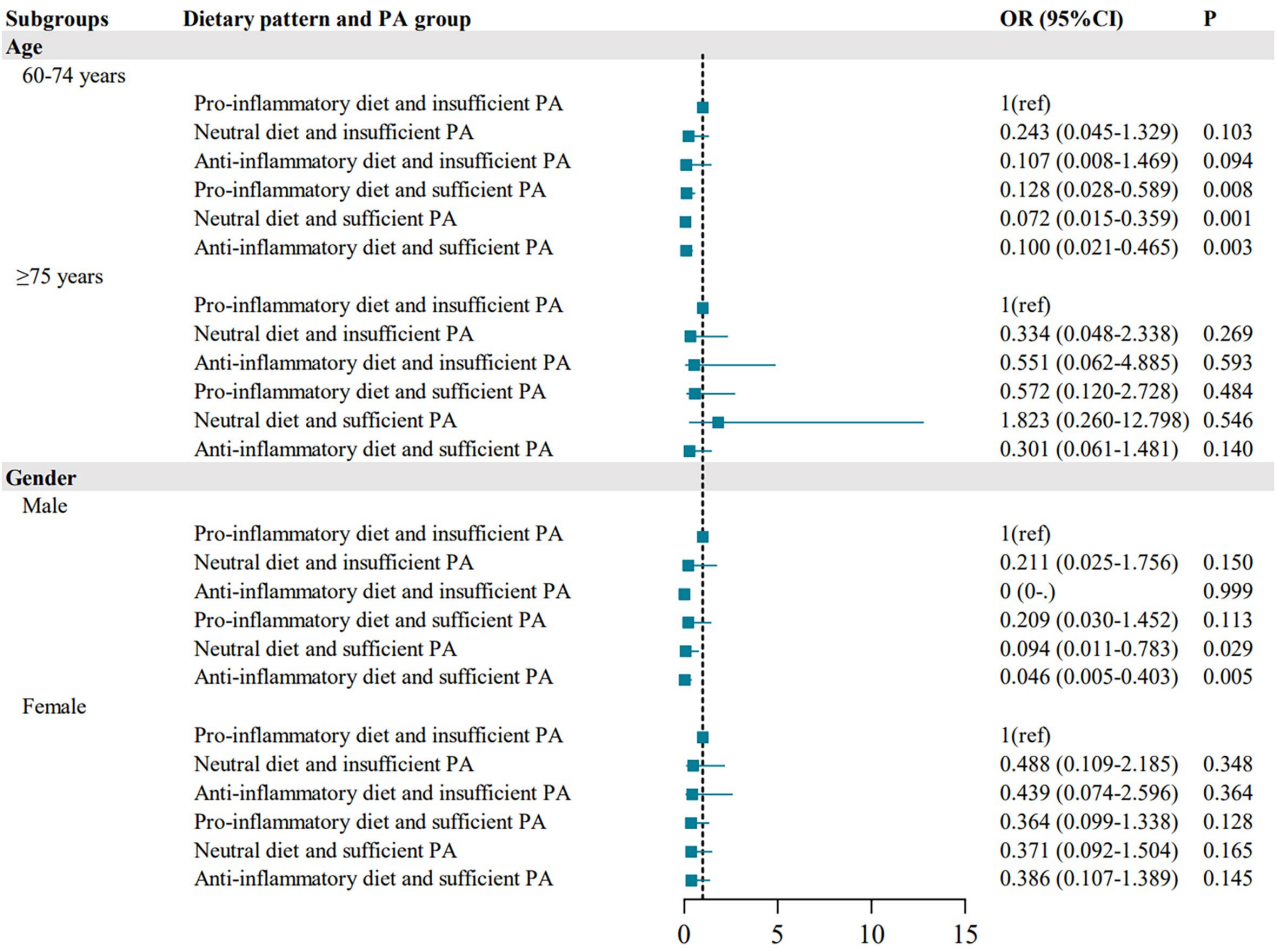
Stratified analyses of the association between dietary patterns and PA with frailty among older adults by different characteristics. ^a^In age subgroup analysis, adjusted for sex, residence area, education level, marital status, smoking, alcohol drinking, and BMI. In sex subgroup analysis, adjusted for age, residence area, education level, marital status, smoking, alcohol drinking, and BMI.

We analyzed the distribution of frailty across different age and gender subgroups within various dietary patterns and PA groups. The results showed that, compared to the pro-inflammatory diet and insufficient PA group, the anti-inflammatory diet and sufficient PA group demonstrated a more pronounced protective effect on frailty in the following subgroups: individuals aged 60–74 years (OR =0.100, 95% CI = 0.021–0.465), males (OR = 0.046, 95% CI = 0.005–0.403). However, no significant protective effect was observed in individuals aged ≥75 years or females. (Figure 1).

### 3.6 Sensitivity analysis of the combined association between dietary patterns, PA, and frailty

**Figure 2:**
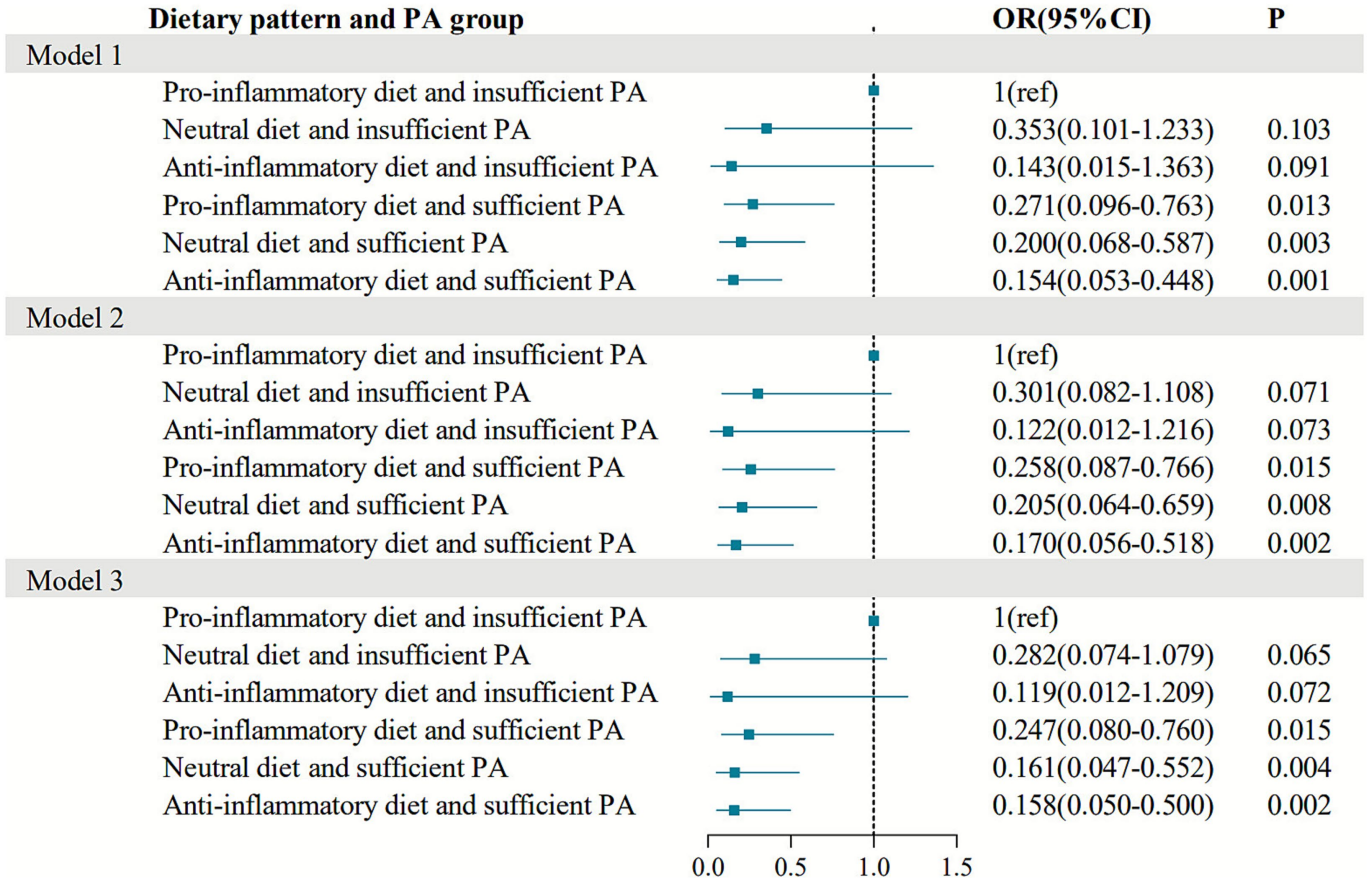
Sensitivity analysis of the combined association between dietary patterns, PA, and frailty. ^a^In age subgroup analysis, adjusted for sex, residence area, education level, marital status, smoking, alcohol drinking, and BMI. In sex subgroup analysis, adjusted for age, residence area, education level, marital status, smoking, alcohol drinking, and BMI.

After excluding 24 older adult individuals aged 80 years and older, a total of 249 participants were included in the final analysis, with 198 non-frail individuals and 51 frail individuals. Logistic regression models were used for the sensitivity analysis. After adjusting for age, gender, residence area, education level, marital status smoking, alcohol drinking, and BMI (Model 3), the results showed that, the following groups were associated with significantly lower odds of frailty compared to the pro-inflammatory diet and insufficient PA group: the pro-inflammatory diet and sufficient PA group (OR = 0.247, 95% CI = 0.080–0.760), the neutral diet and sufficient PA group (OR = 0.161, 95% CI = 0.047–0.552), and the anti-inflammatory diet and sufficient PA group (OR = 0.158, 95% CI = 0.050–0.500) (Figure 2).

## 4 Discussion

In this study, we not only confirmed the respective inverse associations of an anti-inflammatory diet and sufficient PA with frailty in older adults but also, for the first time in a Chinese older adult population, systematically evaluated their combined effect. We found that, compared with the pro-inflammatory diet and insufficient PA group, older adults adhering to an anti-inflammatory diet combined with sufficient PA were associated with significantly lower odds of frailty. Importantly, our results suggest that sufficient PA may partially counteract the potential adverse effects of a pro-inflammatory dietary pattern, demonstrating a compensatory or additive protective effect. This finding provides new empirical evidence for the development of integrated and personalized strategies for the prevention of frailty.

We followed the recommendations of the WHO Guidelines on Physical Activity and Sedentary Behavior for PA levels, and categorized dietary patterns into three groups based on DII to evaluate the combined association of PA and dietary patterns with frailty among 273 older adults. Our findings revealed that, compared with pro-inflammatory diets, adherence to anti-inflammatory diets was associated with a lower likelihood of frailty. Consistent with our results, Shivappa et al. reported that individuals with higher DII scores had a 37% greater risk of frailty compared to those with lower scores (9). Similarly, Chuang et al., in a study of 5,663 adults aged ≥55 years, concluded that pro-inflammatory diets were significantly associated with an increased likelihood of frailty (10). Using different approaches, Qi et al. classified a simplified food frequency questionnaire to classify dietary patterns into plant-based and animal-based diets (23), while He et al. applied the Healthy Eating Index 2015 (HEI-2015) to examine the relationship between diet quality and frailty (24). These differences may stem from the distinct objectives of each study. Regarding PA, our results indicated that, compared with insufficient PA, sufficient PA was associated with a reduced risk of frailty among older adults. This aligns with the findings of Fung et al., which demonstrated that increasing PA levels significantly lowered frailty risk (8). Similarly, Wanigatunga et al., using accelerometer-based measurements among 638 older adults, observed that every additional 30 min of activity reduced the odds of frailty by 10% (25). In contrast, Gil-Salcedo et al., analyzing 6,357 participants, found that only high-intensity activity was effective in reducing frailty risk (26). This discrepancy may stem from differences in study populations, as their participants were aged ≥50 years, whereas our cohort included individuals aged ≥60 years. A study by Umegaki et al. (27) reported findings somewhat different from ours, indicating that older adults with low physical activity demonstrated better cognitive performance relative to frailty compared to those with moderate-to-high physical activity. This discrepancy may be attributable to differences in participant selection criteria. Our study included adults aged 60 years and above, whereas the aforementioned study enrolled individuals aged 65–85 years and might have included a higher proportion of participants already in poorer health. Such fundamental differences in population characteristics may explain the variation in outcomes. Furthermore, research by Resciniti, N. V. et al., which categorized dietary patterns into five groups using the DII, reported that individuals in DII quintile 5 (vs. Q1) were more likely to be frail (OR = 1.70) (28). This finding differs slightly from our results, which suggested a stronger protective benefit of an anti-inflammatory dietary pattern compared to a pro-inflammatory one. Possible reasons for this divergence include the larger sample size in their study and their use of a modified Fried frailty criterion within the NHANES dataset, which may have led to subtle differences in the estimated associations. Although previous research has established the independent associations of PA and diet with frailty, our study extends the existing evidence by demonstrating that, after adjusting for potential confounders, older adults with both anti-inflammatory dietary patterns and sufficient PA exhibited substantially lower odds of frailty compared to those with pro-inflammatory diets and insufficient PA. These findings provide novel evidence for the synergistic role of dietary patterns and PA in mitigating frailty among older adults.

Both anti-inflammatory dietary patterns and sufficient PA are associated with a lower likelihood of frailty in older adults (29–31). These two modifiable lifestyle factors may work synergistically or complementarily through shared biological pathways to mitigate the progression of frailty. At the molecular level, anti-inflammatory diets, rich in polyphenols, dietary fiber, and antioxidant nutrients such as vitamins C and E, can downregulate systemic inflammatory markers including CRP, IL-6, and TNF-*α*, thereby alleviating the physiological burden of “inflammaging” (11, 32). Concurrently, sufficient physical activity not only independently reduces chronic inflammation (33, 34), but also counteracts oxidative stress and metabolic dysfunction by enhancing insulin-like growth factor-1 signaling and improving insulin sensitivity (35, 36). Importantly, the two may establish a positive feedback loop along the “anti-inflammatory–anabolic” axis: anti-inflammatory diets provide high-quality substrates such as leucine to support muscle repair and protein synthesis while inhibiting catabolic signaling pathways (37, 38); meanwhile, PA directly stimulates muscle protein synthesis, promotes neuromuscular adaptation, and improves coordination (39, 40), thereby more effectively preserving skeletal muscle mass and function and delaying the progression of sarcopenia and functional decline (41). Furthermore, these two lifestyle factors may exert additive or synergistic effects through shared mechanisms such as modulating gut microbiota composition, maintaining redox balance, and improving systemic acid–base homeostasis (42, 43). For instance, dietary fiber and polyphenols in anti-inflammatory diets help shape an anti-inflammatory gut microbial profile, whereas PA has been shown to increase gut microbial diversity and enhance intestinal barrier function. This multi-target, multi-pathway interaction may collectively reduce inflammatory load, enhance tissue repair capacity, and improve physiological resilience, thereby more comprehensively preserving physiological reserves in older adults and contributing to a lower likelihood of frailty. Thus, although adopting either an anti-inflammatory diet or sufficient PA alone has demonstrated protective effects against frailty, their combination may yield integrative benefits greater than the sum of their individual effects (“1 + 1 > 2”) in terms of reducing inflammation, promoting anabolism, and optimizing the internal microenvironment. This suggests that frailty-prevention strategies should advocate for the integrated application of nutritional and exercise interventions. Particularly in resource-limited settings or for individuals constrained in one aspect, strengthening the other health behavior may provide partial compensation, paving the way toward a more resilient and personalized path to healthy aging.

In summary, both sufficient PA and anti-inflammatory dietary patterns can effectively reduce the incidence of frailty in the older adults. This study further demonstrates that the pro-inflammatory diet and sufficient PA group, the neutral diet and sufficient PA group, and the anti-inflammatory diet and sufficient PA group were all significantly associated with lower odds of frailty. The results suggest that sufficient PA can effectively prevent frailty in older adults, regardless of whether an anti-inflammatory, neutral, or pro-inflammatory dietary pattern is adopted. These findings may indicate that the preventive and intervention effects of PA on frailty in older adults may outweigh the benefits derived from diet alone. PA may even counteract the potential negative impact of a pro-inflammatory dietary pattern on frailty. The physiological mechanisms underlying this could be related to PA’s direct downregulation of inflammation, its effective promotion of muscle synthesis, and its overall optimization of the neuromuscular system. This discovery holds significant public health implications: it suggests that, within resource-limited health promotion strategies, prioritizing and encouraging regular PA for older adults could be a more efficient and universally applicable approach than simply advocating for specific dietary patterns. However, this does not diminish the importance of a healthy diet, as the benefits of anti-inflammatory diets for overall health have been widely acknowledged. Moreover, some older adult individuals may be limited by physical health conditions or other factors, preventing them from maintaining sufficient PA. For such individuals, adopting an anti-inflammatory diet may be an effective strategy to help mitigate frailty. Future research should exploring the specific physiological mechanisms behind the interactions between PA and different dietary patterns, and develop more personalized “activity-diet” integrated interventions for older adults with varying health statuses.

We also found that, among both younger (60–74 years) and older (≥75 years) adults who adhered to an anti-inflammatory diet and sufficient PA, the younger group was associated with a lower prevalence of frailty. This may be due to the fact that younger older adult individuals generally have better physical function reserves and greater plasticity. Adhering to an anti-inflammatory diet and regular PA can effectively prevent the onset of frailty and even reverse early frailty (44). In contrast, older the oldest adult individuals tend to experience more severe “inflammatory aging,” multimorbidity, significantly reduced physiological reserves, higher rates of malnutrition, and sarcopenia (35, 38). For this group, a single intervention is more likely to delay functional decline and maintain existing capabilities rather than result in significant improvement, thus requiring more intensive and comprehensive interventions. In older adults of different genders, adherence to an anti-inflammatory diet and sufficient PA was more effective in preventing frailty in men than in women. This may be due to the fact that older women are more prone to falls, osteoporosis, and fractures compared to men. Additionally, the reduction in hormone levels in older women leads to increased chronic inflammation and elevated levels of C-reactive protein and IL-6, which exacerbate the inflammatory state and impair muscle function (45). Therefore, under the same conditions of following an anti-inflammatory diet and engaging in sufficient PA, the incidence of frailty in men was significantly lower than in women.

This study has several limitations. First, our research is a cross-sectional study, which may not establish a definitive causal relationship between dietary patterns, PA and frailty. Future studies should use prospective cohort designs or randomized controlled trials to verify these findings. Second, the sample size in this study is relatively limited and drawn from a specific region, which may introduce selection bias. Future studies could expand the sample size and implement multi-center sampling to improve the generalizability of the results. Third, there are two potential methodological limitations in the data collection process of this study. When using FFQ25 to assess dietary intake in older adults, recall bias may have occurred among respondents, which could affect the accuracy of the calculated dietary inflammation index. Additionally, the accelerometer used in this study could not effectively record data on aquatic activities (such as swimming), which may lead to measurement bias in the overall assessment of PA levels. Future studies should combine biomarkers with multi-source motion sensors to complement the recall bias of questionnaires and the limitations of accelerometers in monitoring aquatic activities, thereby improving data accuracy. Authors should discuss the results and how they can be interpreted from the perspective of previous studies and of the working hypotheses. The findings and their implications should be discussed in the broadest context possible. Future research directions may also be highlighted.

## 5 Conclusion

Our study shows that, compared to older adult individuals with a pro-inflammatory diet and insufficient PA, those following an anti-inflammatory diet and engaging in sufficient PA were associated with significantly lower odds of frailty. Moreover, the role of PA in preventing frailty may outweigh that of dietary interventions and can partially counteract the negative effects of a pro-inflammatory diet. These findings offer new insights and strategies for preventing and managing frailty in older adults.

## Data Availability

The datasets presented in this study can be found in online repositories. The names of the repository/repositories and accession number(s) can be found in the article/supplementary material.
